# The effect of telehealth-based medical nutrition therapy on cardiovascular disease risk factors in a rural population: a secondary analysis of outcomes related to nutrition, health and well-being from the healthy rural hearts randomised controlled trial

**DOI:** 10.1186/s12966-025-01819-3

**Published:** 2025-10-13

**Authors:** Tracy L. Schumacher, Erin D. Clarke, Jaimee Herbert, Anna Jansson, Chris Oldmeadow, Megan E. Rollo, Penny Milson, Carissa Alderton, Leanne J. Brown, Jennifer May, Annabelle Williams, Michelle Guppy, Shanthi Ramanathan, John Attia, Clare E. Collins

**Affiliations:** 1 Department of Rural Health, College of Health Medicine and Wellbeing, University of Newcastle, Tamworth, NSW 2340 Australia; 2 Food and Nutrition Program, Hunter Medical Research Institute, New Lambton Heights, NSW 2305 Australia; 3 School of Health Sciences, College of Health Medicine and Wellbeing, University of Newcastle, Callaghan, NSW 2308 Australia; 4 Data Sciences, Hunter Medical Research Institute, New Lambton Heights, NSW 2305 Australia; 5 School of Population Health, Faculty of Health Sciences, Curtin University, Bentley, WA 6102 Australia; 6 Hunter New England Central Coast Primary Health Network, Tamworth, 2340 Australia; 7 School of Rural Medicine, University of New England, Armidale, NSW 2350 Australia; 8 School of Medicine and Public Health, University of Newcastle, College of Health Medicine and Wellbeing, Callaghan, NSW 2308 Australia; 9 Research Impact Platform, Hunter Medical Research Institute, New Lambton Heights, NSW 2305 Australia

**Keywords:** Medical nutrition therapy, Primary care, Cardiovascular disease, Rural, Telehealth, Dietitian, Randomised controlled trial, Diet

## Abstract

**Background:**

Adults in rural Australia are at elevated risk of cardiovascular disease (CVD). To date, no intervention trials have evaluated the impact of dietitian delivered nutrition therapy using telehealth exclusively in patients recruited in the primary care setting. The primary aim was to assess effectiveness of telehealth delivered Medical Nutrition Therapy (MNT) on change in dietary intake energy, reported as percent energy derived from nutrient-dense (core) foods. Secondary aims included assessment of the intervention effects on percentage weight loss, quality of life, health literacy and patient engagement in their health (patient activation).

**Methods:**

This was a secondary data analysis of a pragmatic cluster Randomised Controlled Trial (RCT). Adults from rural areas within the New England North West and Upper Hunter regions of New South Wales, Australia, were identified by their general practitioner (GP) following a Heart Health Check, as being at moderate-to-high risk of CVD and invited to participate. General practices were randomised into intervention or usual care groups. Intervention participants received five personalised telehealth MNT consultations over 6 months. Usual care received stand-alone personalised nutrition reports. All participants were managed by their GP and followed up after 12 months. Primary and secondary outcomes were analysed using Bayesian linear mixed models. Models included fixed categorical effects for time, group, group-by-time interaction, age, and sex, with additional predetermined adjustment for variables determined by the literature.

**Results:**

Mean baseline to 12-month increase in percentage of energy from core foods was 7.0% (9.4 SD) for the intervention group and 1.3% (9.6 SD) for usual care group, with an estimated adjusted difference in mean change of 5.9% (95%CI 0.5–11.2). Significant improvements in quality of life (0.04, 95%CI 0.01–0.07) and patient activation were also observed (6.44, 95%CI 0.99–11.83) favouring the intervention group.

**Conclusion:**

A personalised telehealth MNT intervention delivered by dietitians significantly improved percentage energy from nutrient-dense foods amongst rural adults at an elevated risk of CVD. Future research is required to support implementation of telehealth MNT into general practice in rural Australia.

**Trial registration:**

Australian New Zealand Clinical Trials Registry (ACTRN12621001495819).

**Supplementary Information:**

The online version contains supplementary material available at 10.1186/s12966-025-01819-3.

## Background

Despite rural populations reporting higher levels of wellbeing and life satisfaction compared to their metropolitan counterparts [1], living in rural locations has been associated with socio-economic disadvantage and increased premature cardiovascular disease (CVD) morbidity and mortality [2, 3]. Specifically, many people living in rural areas of Australia have increased risk of CVD [2]. In the rural New England North West region of New South Wales in Australia, where this study was conducted, the age standardised rate of deaths related to coronary heart disease was 60.4 per 100,000 people compared to 52.8 per 100,000 people nationally between 2018 and 2022 [4]. Challenges in rural areas that can exacerbate health inequities and contribute to higher CVD prevalence include having to travel longer distances to receive health care, lower ratios of and higher turnover of healthcare staff, higher consultation costs, and delays in accessing timely care [5–7]. However, despite clear health disparities, rural populations have been underrepresented in Australian health-related research [8]. Australian policy makers acknowledge that there is a lack of evidence to guide strategies to address this higher CVD burden, applicable to rural Australia regarding the burden of CVD [9].

Telehealth has the potential to reduce health access inequalities for CVD prevention and treatment in rural areas by improving access to dietetics as part of primary care services [7, 10]. Dietitian-led interventions, including those with a behaviour change component, have evidence from systematic reviews and meta-analyses for improved cardiovascular related risk factors, such as weight management, waist circumference, blood pressure, lipids, and blood glucose [11–15]. These risk factors can be addressed through a focus on food groups and eating patterns that align with national and international recommendations and guidelines [13, 16–19]. These emphasise increasing intakes of nutrient-dense core foods, such as fruits, vegetables, legumes, wholegrains, lean protein and dairy foods, while limiting nutrient-poor foods that are high in energy, saturated fat, sodium, and/or added sugars [13, 16–19]. Adherence to dietary guidelines and heart healthy diet guidelines has been demonstrated to reduce relative risk of CVD by 14–19% [20]. While the evidence is promising for personalised medical nutritional therapy (MNT) for managing CVD risk factors in people at elevated CVD risk, limited studies have applied this in rural Australia and even fewer studies have reported using telehealth to implement interventions [9, 21].

Evidence suggests an interplay between dietary patterns and quality of life (QOL), which may impact CVD outcomes [22]. This may have implications for patient CVD outcomes, as lower health related QOL has been shown to be significantly associated with adverse CVD outcomes, including increased risk of hospitalisation and mortality, even after adjusting for health behaviours, comorbidities and demographics such as age and sex [23, 24]. Associations between QOL and self-efficacy have also been found in CVD populations, indicating a relationship between a person’s confidence in their ability to carry out tasks and how they feel physically, mentally, socially and functionally [25]. However, most of the data relating QOL, diet quality and CVD outcomes originate from observational studies. Therefore, it is important to evaluate the relationship between dietary intake, QOL and CVD risk factors in intervention studies in at risk populations.

Patient ‘activation’ and health literacy levels have also been found to impact CVD outcomes. Compared to those less engaged, patients with high levels of ‘activation’ who choose to be engaged and take an active role in managing their health, have better healthcare outcomes, including CVD outcomes [26–28]. Conversely, lower health literacy has been found to have adverse impacts on mortality and hospital admissions related to CVD [29], as well as being associated with unhealthy lifestyle behaviours such as smoking, inadequate physical activity [30–32] and in some cases poorer diet quality [33]. Healthcare providers can facilitate patient activation by considering their patients' health literacy, and adopting a collaborative approach to health management that involves their patients in the creation of their health management plans [33]. Despite the active role dietitians have in providing MNT, very few studies have investigated dietitians impact on patient activation and health literacy [11–15, 31]. This highlights a gap in understanding relating to MNT practice and research.

The primary aim of the current study was to assess the change in percentage of energy from nutrient-dense core foods, after a 12-month personalised, dietary intervention delivered by telehealth for rural Australians assessed as at moderate to high risk of CVD by their primary care physician or general practitioner (GP), as commonly known in Australia. Secondary aims included assessment of change in QOL, patient activation measures and health literacy over the 12-month intervention period.

## Methods

This paper reports on nutrition, health and well-being outcomes as an exploratory secondary analysis of results from Healthy Rural Hearts (HealthyRHearts), a 12-month pragmatic cluster randomized controlled trial that compared two levels of dietary advice provided to participants at an elevated risk of CVD. The study took place within primary care settings in rural New South Wales, Australia, and was registered with Australian New Zealand Clinical Trials (ACTRN:12,621,001,495,819). It received ethics approval from the University of Newcastle Human Research Ethics Committee (H-2021-0193) and safety approvals from the University of Newcastle Health and Safety Committee (49/2021). Full details relating to the study methods and nutrition intervention protocol have been published in detail elsewhere [34, 35]. Results from the primary outcomes of this intervention found no difference between groups at 12 months for total cholesterol or blood pressure, although findings indicated improvements in blood glucose control [36]. This analysis is reported according to the 2010 Consort extension statement for cluster randomised trials [37].

### Participants

Recruitment took place in two stages. In Stage 1 between February 2022 and March 2023, primary care practices and GPs were recruited to the study. To be eligible, primary care practices had to be based within the Hunter New England Central Coast Primary Health Network (HNECC PHN) region, categorised as a rural or remote location according to the Modified Monash Model (MM) 3–7 [38], and have a data sharing agreement with the HNECC PHN. At the time of recruitment planning (9th Feb 2021), 76.8% of primary care practices met this criterion, although no practices existed in MM 6–7. Therefore, all practices were located in areas described as large (MM 3), medium (MM 4) or small rural towns (MM 5). In Stage 2, consenting primary care practices and GPs recruited participants using study-provided materials, including an invitation letter signed by the GP, a study pamphlet, participant information statement, consent form and reply-paid envelope for return to the research team. Participants were recruited between February 2022 and July 2023, with the final 12-month data obtained in July 2024. Recruitment was halted to ensure final data for all participants could be included in the final analysis that coincided with the funding period. To be eligible, participants had to be assessed by their GP as being at moderate-to-high risk of a CVD event (≥ 10%) within the next 5 years, using the 2012 CVD risk calculator [39] (based on the Framingham risk equation), or using clinical judgment and referring to the Guidelines for the management of absolute cardiovascular disease risk [40]. People were ineligible to participate if they had a medical condition that significantly affected dietary intake, had recent change in treatment/s where effectiveness had not yet stabilised (such as coronary revascularization in the last 6 months or a change in statin medication in the preceding 3 months), had no email address or access to the internet.

### Randomisation and blinding

A block randomisation sequence, based on the primary care practice, was generated by a statistician external to the study and uploaded to REDCap, the software tool used to collect and manage study data [41, 42]. Primary care practices were stratified by MM categories (3, 4 and 5 only, as no practices based in the specified area were category 6–7), and practice size (small: 1–5 GPs, medium: more than 5 GPs) to the intervention or usual care group. Practices were assigned to a group after the first patient invitation was posted. Practices and GPs were informed of their allocation by research staff, meaning they could provide appropriate care with the knowledge of whether their patient was receiving a dietetic intervention or not.

### Interventions

#### Usual care arm

Usual care consisted of care as deemed appropriate by the participant’s GP. No restrictions were placed on changes to medication or referrals to other services. At the end of the 12-month usual care allocation, participants were invited to return to their GP for an annual heart health check. Additionally, those in the usual care arm were offered two consultations (approximately one hour) with a study dietitian over a two-week period following their completion of the 12-month allocation.

Usual care was also inclusive of being invited to complete the Australian Eating Survey (Heart version, AES-Heart) [43–45] at baseline, 3-, 6- and 12-month time points. The AES-Heart is an extended version of the online adult AES Food Frequency Questionnaire and captures additional details about foods or nutrients evidenced to be consistent with good heart health [13, 16]. When participants completed the AES-Heart they received an immediate personalized nutrition report based on their dietary intake that compared intake to recommendations in the Australian Dietary Guidelines and the Heart Foundation evidence-based Heart Healthy eating principles [13, 16]. Collectively, these principles and recommendations focus on increasing intakes of nutrient-dense core foods and decreasing non-core, energy-dense foods. Nutrient-dense core-foods were defined as foods that align with recommendations and include fruits, vegetables, wholegrains, healthy protein rich foods, dairy, healthy fats and oils, soy foods, nuts and legumes [13, 16]. Non-core foods were defined as energy-dense and nutrient-poor as per the Australian Guide to Healthy Eating [16], and are foods high in saturated fat, sodium and/or added sugars. An example of a personalized AES-Heart report is provided in Supplementary materials 1.

#### Intervention arm

In addition to usual care, participants randomized to the intervention group were allocated five telehealth MNT consultations with an Accredited Practising Dietitian (APD) (the Australian equivalent of a Registered Dietitian) over six months [46]. APDs additionally received training specific to the study, which included patient-centred counselling strategies [35]. Dietetic consultations occurred at baseline (30 min), 2-weeks (20 min), 4-weeks (20 min), 3-months (20 min) and 6-months (30 min) and used HealthDirect, a telehealth video consultation platform that meets Australian privacy requirements [47].

Michie’s behaviour change paradigm was the theoretical framework that underpinned the MNT behaviour change consultations [48]. To assist with implementing this behaviour change framework in practice, participants completed an online version of the Personalised Nutrition Questionnaire (PNQ). This operationalised the behaviour change component of capability, opportunity, motivation and behaviour (COM-B) characteristics [48, 49]. Participant PNQ responses were used together with their individualised AES-Heart reports to provide personalised, tailored nutrition resources that targeted CVD prevention. The nature of the dietary counselling was at the professional discretion of the APD, who based their advice on their patient-centred counselling training, participant goals, PNQ results, and evidenced-based dietary patterns and national guidelines [13, 16]. Detailed information about the development and structure of the MNT intervention has been published elsewhere [34, 35].

The primary outcome measure was percentage energy from nutrient-dense core foods, i.e. total consumption of foods consistent with evidence-based Heart Healthy eating principles [13, 16]. Energy and nutrient values were derived from the AES-Heart, based on adult portion sizes and frequencies of intake reported from the food list. Nutrient analysis of core and non-core foods used the AUSNUT 2011–13 food and nutrition database [50]. Estimated intake per day of macronutrients, micronutrients and food group serves (e.g. fruit, vegetables) were calculated from individual food list items and reported for baseline only to allow comparison with other populations.

Secondary outcomes included QOL, patient activation measure (PAM), weight loss and health literacy measures. Weight loss was reported as a binary outcome and limited to whether a person achieved a clinically relevant 5% weight loss [51]. Details related to outcome measures and methodology can be found in Supplementary materials 2: Outcome measures. Other factors that may impact on dietary intake, such as quality of sleep and physical activity, were also collected and reported for baseline only.

### Statistical analysis

This is an exploratory secondary outcomes data analysis of the HealthyRHearts study [34]. The primary study was powered to detect a 0.51 change in total cholesterol, and was affected by COVID-19, resulting in a reduced sample size, inconsistent patient numbers from practice clusters, and a 15-month extension to the estimated 24-month trial. To account for the reduced sample size, a Bayesian approach was used for the analysis, consistent with other trials where recruitment was adversely impacted by the COVID-19 pandemic [52]. Since a non-significant p-value implies the absence of an effect, we have used a Bayesian approach which allows for meaningful inference even with smaller sample sizes, rather than provide inconclusive results due to a lack of power.

Demographic and descriptive statistics were performed using Stata/IC v16.1. The mean change from baseline in the primary and secondary outcomes were compared between groups using Bayesian linear mixed models in R (v4.3.2). Models included fixed categorical effects for time, group, group by time interaction, age, and sex. Missing data was assumed to be missing at random. Other variables adjusted for in outcome measures were predetermined from the literature. The model included random intercepts for individual participants to model repeated measures over time, and the primary care practice from which they were recruited, to account for the cluster randomized design. Estimates from the joint posterior distribution were obtained through Markov chain Monte Carlo (MCMC) sampling (the No- = UTurn-sampler, implemented in the brms package with four chains of 10,000 iterations and a warmup of 1000. Flat priors were used for all fixed effects, a student t (location = 0.00, scale = 2.50) for the error standard deviation. Priors over the random effect standard deviation for individuals and GPs were set as cauchy (location = 0, scale = 5) and cauchy (location = 0, scale = 2) respectively. Convergence and stability of the Bayesian sampling has been assessed using R-hat, which should be below 1.01 [53] and Effective Sample Size (ESS), which should be greater than 1000 [54]. Model fit was assessed by taking 10 random draws from the posterior predictive distribution compared against the observed outcome distribution. Mean posterior estimates are presented, together with 95% highest density credible intervals, the probability the parameter is greater than zero for positive estimates, or less than zero for negative estimates (the probability of direction (pd)) and the evidence ratio (pd/1-pd).

## Results

**Fig. 1:**
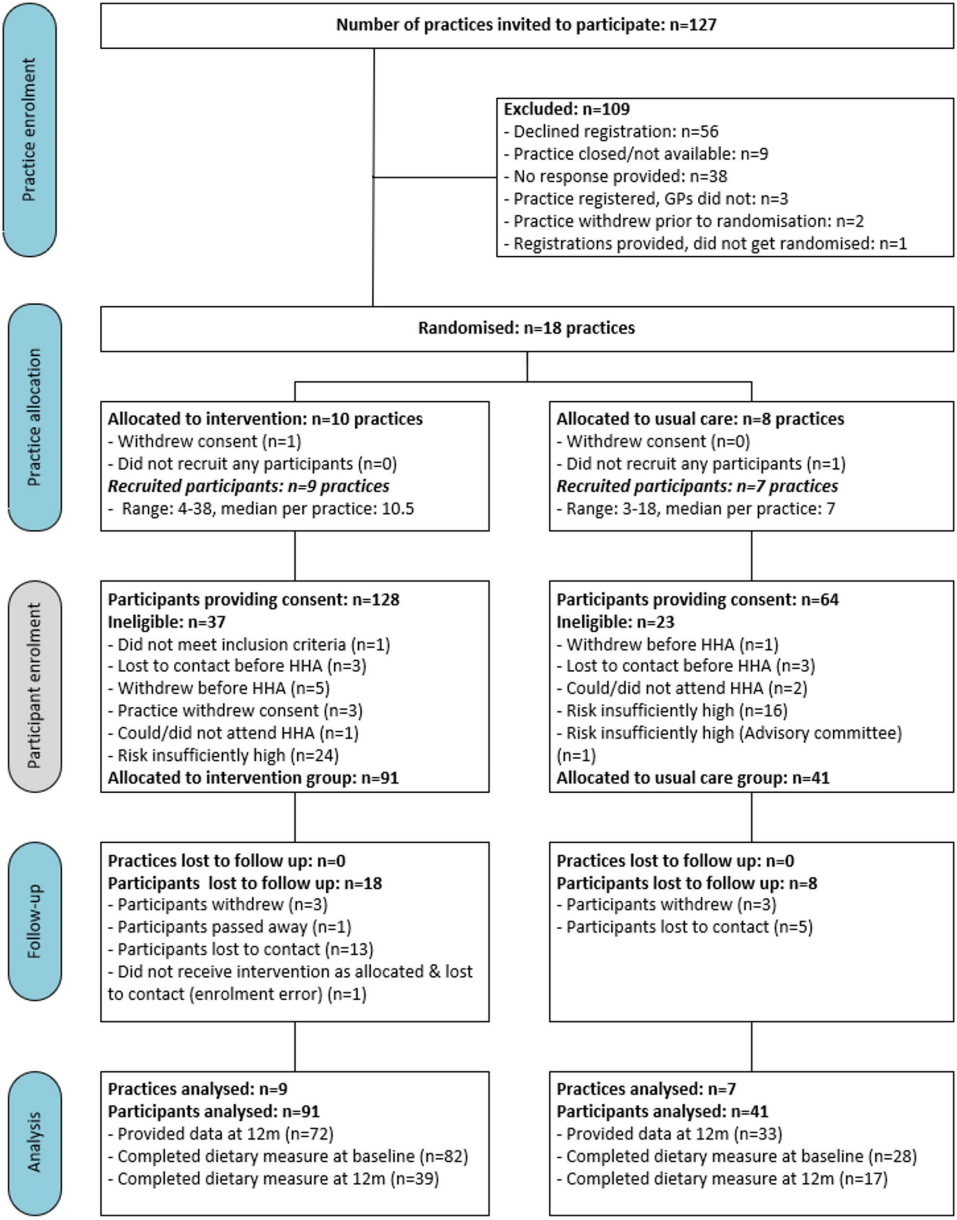
Healthy Rural Hearts Participant Flowchart. HHA: Heart Health Assessment

All eligible practices in the geographical study area were invited to participate (n = 127), with consent received from 18 primary care practices (Fig. 1). One practice withdrew consent prior to assessing participants for eligibility, due to time constraints. One usual care practice could not recruit any participants, leaving nine practices randomised to the intervention group and seven to usual care that contributed participants. Consent was received from 192 patients, with 173 being assessed for cardiovascular risk. Following assessment, 91 patients were enrolled from intervention practices and 41 from usual care. Completion rates were approximately equal in both groups (Int: n = 72/91 and UC: n = 33/41).

Attendance rates at MNT consultations were initially high, with 96% (n = 87/91) of intervention participants attending their baseline consultation. An error led to an incorrect enrolment for one participant, who did not receive the MNT consultations as described. Participant attendance declined over the subsequent consultations, with 91% attendance at both 2- and 4-weeks (n = 83), 79% at 3-months (n = 72) and 70% of intervention participants completing MNT consultations at 6-months (n = 64). At 12 months, 49/91 intervention and 21/41 UC participants returned data for PAM and QOL measures. The same number of responses were obtained for both health literacy values of AAHLS and eHEALS (Int = 49/91 and UC = 20/41). A weight value was obtained from Int = 68/91 and UC = 28/41, by compiling participant self-report and GP collected measures.

Table 1 summarizes the baseline characteristics of included participants and Table 2 shows baseline dietary intake. While some differences were observed between groups at baseline, these were accounted for in the statistical models.

**Table 1:** Demographic characteristics of the Healthy Rural Hearts Participants (n = 132)

	Reference Range	Intervention (n = 91)	Usual Care (n = 41)	Total Sample (n = 132)
Age (years) (mean ± SD)		63.8 ± 5.6	61.3 ± 5.0	63.1 ± 5.5
Sex (Females, n (%))		27 (29.7%)	17 (41.5%)	44 (33.3%)
BMI (kg/m^2^)		31.6 ± 6.8	32.1 ± 5.4	31.7 ± 6.4
(missing)		4 (4.4%)	5 (12.2%)	9 (6.8%)
*Self-reported demographics*				
Health conditions n (%)^a^				
High cholesterol		49 (53.9%)	29 (70.7%)	78 (59.1%)
High blood pressure		57 (62.3%)	29 (70.7%)	86 (65.2%)
Diabetes		19 (20.9%)	10 (24.4%)	29 (22.0%)
Overweight or obesity		37 (40.7%)	21 (51.2%)	58 (43.9%)
(missing)		8 (8.8%)	5 (12.2%)	13 (9.9%)
Rural residence classification^b^				
MM 2/3		29 (31.9%)	15 (36.6%)	44 (33.3%)
MM 4		30 (33.0%)	18 (43.9%)	48 (36.4%)
MM 5/6		32 (35.2%)	8 (19.5%)	40 (30.3%)
Has private health insurance		46 (50.6%)	25 (61.0%)	71 (53.8%)
(missing/don’t wish to answer)		4 (4.4%)	1 (2.4%)	5 (3.8%)
Education level				
Year 12 or less		43 (47.3%)	16 (39.0%)	59 (44.7%)
Trade or vocation		20 (22.0%)	12 (29.3%)	32 (24.2%)
University or higher		20 (22.0%)	8 (19.5%)	28 (21.2%)
(missing)		8 (8.8%)	5 (12.2%)	13 (9.9%)
Household income ($AUD per week)				
< $700		14 (15.4%)	7 (17.1%)	21 (15.9%)
$700-$999		19 (20.9%)	6 (14.6%)	25 (18.9%)
$1,000-$1,499		15 (16.5%)	4 (9.8%)	19 (14.4%)
$1,500-$2,499		13 (14.3%)	6 (14.6%)	19 (14.4%)
$2,500 or higher		10 (11.0%)	10 (24.4%)	20 (15.2%)
(missing / don’t wish to answer)		20 (22.0%)	8 (19.5%)	28 (21.2%)
Sleep (t-score)^c^	28.9–76.5	51.8 ± 2.7	51.4 ± 4.5	51.7 ± 3.3
(missing)		9 (9.9%)	6 (14.6%)	15 (11.4%)
Quality of life (EQ-5D)^d^	0.0–1.0	0.68 ± 0.09	0.68 ± 0.09	0.68 ± 0.09
(missing)		9 (9.9%)	6 (14.6%)	15 (11.4%)
Physical activity (median (IQR))	Minutes per week	375 (180–760)	415 (140–870)	390 (150–780)
(missing)		9 (9.9%)	7 (17.1%)	16 (12.1%)
Health literacy (eHEALS)^d^	8–40	24.9 ± 6.6	25.2 ± 6.4	24.9 ± 6.5
(missing)		9 (9.9%)	6 (14.6%)	15 (11.4%)
Health literacy (AAHLS)^d^	11–33	24.9 ± 3.1	24.3 ± 2.7	24.6 ± 3.0
(missing)		9 (9.9%)	6 (14.6%)	15 (11.4%)
Patient activation measure^d^	0–100	57.0 ± 10.3	59.4 ± 12.5	57.7 ± 11.0
(missing)		8 (8.8%)	6 (14.6%)	14 (10.6%)
Average number nutrition goals set at baseline^e^		1.9 ± 0.8	-	-

^a^Assessed by asking participants if they had been told by a health care professional that they have any of the listed conditions

^b^E.g.: MM 2: Regional centre; MM 3 large rural town (population 15,000 to 50,000); MM 4 medium rural town (population 5,000 to 15,000); MM 5 small rural town; MM 6 remote community. Note: MM 2/3 and MM 5/6 have been combined to protect participant anonymity, due to limited numbers in MM 2 and MM 6

^c^Assessed using the PROMIS short form Sleep Disturbance 4a. A T-score was calculated by rescaling the raw score into a standardised score, using a mean of 50 and a standard deviation of 10

^d^Higher scores are associated with better quality/knowledge

^e^Average of the 86 intervention participants who completed goal setting within their MNT appointment. No data for n = 15

**Table 2:** Baseline nutrition characteristics for the Healthy Rural Hearts participants

	Intervention (n = 82)	Usual Care (n = 28)	Total Sample (n = 110)
	Mean ± SD	Mean ± SD	Mean ± SD
Energy intake (kilocalorie/d)	2651 ± 972	2235 ± 634	2454 ± 914
Energy intake (kilojoule/d)	11,093 ± 4068	9353 ± 2652	10,650 ± 3824
% Energy Core Foods ^(a)^	63.9 ± 12.3	64.1 ± 10.9	63.9 ± 11.9
% Energy Non-Core Foods ^(b)^	36.2 ± 12.3	35.9 ± 10.9	36.1 ± 11.9
*MACRONUTRIENTS & MICRONUTRIENTS*			
% Energy Protein	18.0 ± 3.4	18.1 ± 3.3	18.0 ± 3.3
% Energy Carbohydrates	38.4 ± 7.7	38.5 ± 6.0	38.4 ± 7.3
% Energy Total fat	37.7 ± 7.2	36.9 ± 7.6	37.5 ± 7.2
Saturated	14.2 ± 3.1	13.7 ± 3.0	14.0 ± 3.1
Monounsaturated	15.5 ± 3.9	15.3 ± 4.1	15.5 ± 4.0
Polyunsaturated	5.8 ± 2.0	6.1 ± 2.4	5.9 ± 2.1
	Median (IQR)	Median (IQR)	Median (IQR)
Omega 3 (milligrams/d)	455 (248–766)	397 (277–589)	437 (269–754)
Fibre (g/d)	30.5 (22.9, 41.2)	28.2 (22.7, 33.3)	30.2 (22.7, 38.1)
Alcohol (g/d)	14.4 (1.0, 35.6)	12.7 (0.6, 35.8)	13.8 (0.8, 35.6)
Sodium (mg/d)	2554 (1840, 3079)	2041 (1795, 2393)	2336 (1829, 2874)
% from core foods	43.7 (36.9, 52.2)	46.4 (40.6, 52.4)	44.7 (38.1, 52.3)
% from non-core foods	46.4 (38.1, 52.8)	47.7 (37.5, 52.9)	46.8 (38.0, 52.8)
% from added salt	9.3 (5.7, 12.5)	8.6 (2.8, 12.4)	8.9 (5.6, 12.5)
FOOD GROUP SERVES			
Fruit (serves/d)^(c)^	1.6 (0.6, 2.5)	1.2 (0.8, 1.7)	1.4 (0.6, 2.4)
Vegetables (serves/d)^(d)^	4.6 (2.7, 5.7)	3.7 (2.9, 5.3)	4.5 (2.8, 5.6)
Nuts (serves/d)^(e)^	0.3 (0.1, 0.9)	0.4 (0.1, 0.6)	0.3 (0.1, 0.8)
Fish and seafood (serves/week)^(f)^	1.6 (0.7, 2.7)	1.8 (0.9, 2.6)	1.7 (0.7, 2.7)
Dairy (serves/d)^(g)^	1.2 (0.6, 1.9)	1.0 (0.6, 1.3)	1.3 (0.6, 1.1)
Grains and cereals (serves/d)^(h)^	2.7 (1.5, 4.4)	2.2 (1.8, 3.1)	2.6 (1.6, 3.9)
Red meat (serves/week)^(i)^	5.5 (3.7, 7.7)	5.1 (3.1, 6.9)	(3.6, 7.7)

^(a)^Core foods are nutrient-dense, consisting of five food groups: fruits, vegetables, dairy, grains/cereals, and meat/meat alternatives

^(b)^Non-core foods are energy-dense and nutrient-poor. Example foods include baked sweet products, alcohol, fatty and processed meats, spreads and sauces, fried and takeaway foods and confectionery

^(c)^A serve of fruit is 150g, in Australia recommendations are for 2 serves/d

^(d)^A serve of vegetables is 75g, in Australia recommendations are for 5 + serves/d depending on sex and age

^(e)^A serve of nuts is 30 g/d, in Australia recommendations are for 1 serve/d

^(f)^A serve of fish and seafood is 100g of cooked fish, in Australia recommendations are for 2–3 serves a week

^(g)^A serve of dairy is 250ml milk or calcium fortified milk alternative, 2 slices of cheese or 200g of yoghurt, in Australia recommendations are for 2.5 + serves/d depending on sex and age

^(h)^A serve of grains and cereals is 1 slice of bread, 1/2 cup cooked porridge, rice or pasta or 2/3 cup wheat cereal flakes, in Australia recommendations are for 5 + serves/d depending on sex, age and activity level

A serve of red meat is 65g of cooked meat, in Australia it is recommended to limit red meat consumption to < 350g per week

The mean increase (SD) in percentage energy from core foods from baseline to 12-month follow-up was 1.3 (9.6) for the UC group and 7.0 (9.4) for the intervention group. The estimated adjusted difference in mean change (intervention minus UC) was an increase of 5.9% energy from core foods at 12 months (95%CI 0.5–11.2). The posterior probability that this difference was positive (indicating a greater increase in percentage energy from core foods compared to the UC group) was 98% (see Table 3). This suggests that the increase in percentage energy from core foods peaked at 3 months (7.7, 95%CI 2.3–13.1), following the intensive dietary intervention phase for the intervention group, with a smaller increase at 6 months (7.0, 95%CI 1.5–12.6). Percentage energy was shown to be higher among participants for each additional year of age by approximately 0.6% (0.6, 95%CI 0.1–1.0), and lower in men (−4.6, 95%CI −8.9, −0.0).

**Table 3:** Outcome estimates collected at all timepoints for percentage energy from core foods, quality of life (Global 10/EQ-5D) and PAM were derived from Bayesian linear mixed models, using MCMC sampling with four chains of 10,000 iterations and random intercepts for individuals and primary care practices, and adjusted for any differences between groups

		Percentage energy from core foods		Quality of life (Global 10/EQ-5D)		Patient activation measure (PAM)	
Variable	Level or statistic	Mean est	95% CI	Mean est	95% CI	Mean est	95% CI
Intercept		36.23	8.54, 64.86	0.16	−0.08, 0.40	30.00	7.17, 53.31
Age	Years	**0.57**	**0.13, 0.99**	0.01	0.00, 0.01	**0.45**	**0.08, 0.81**
Gender	Female	REFERENCE		REFERENCE		REFERENCE	
	Male	−**4.55**	−**8.93, **−**0.02**	0.01	−0.02, 0.05	−1.86	−5.89, 2.23
Education	Year 12 or less	REFERENCE					
	Trade or vocation	3.61	−1.16, 8.30	-	-	**4.66**	**0.26, 9.13**
	University or higher	4.62	−0.36, 9.48	-	-	4.50	−0.06, 9.02
Living arrangements	By self	REFERENCE		REFERENCE			
	With partner	−**7.98**	−**14.30, **−**1.58**	−0.02	−0.07, 0.03		
	With children	−10.04	−25.66, 5.66	−0.07	−0.19, 0.05		
	With partner & children	−7.16	−15.80, 1.40	−0.02	−0.09, 0.05		
	Other	−5.60	−16.89, 6.03	−0.01	−0.08, 0.11		
Household income	< $700 p/w	-		REFERENCE			
($AUD)	$700—$999 p/w	-	-	0.04	−0.01, 0.09		
	$1,000—$1,499 p/w	-	-	**0.08**	**0.03, 0.13**		
	$1,500—$2,499 p/w	-	-	0.06	0.00, 0.11		
	> $2,500 p/w	-	-	**0.10**	**0.04, 0.15**		
Time^1^	Baseline	REFERENCE		REFERENCE		REFERENCE	
	3 months	−0.27	−5.07, 4.54	−0.01	−0.04, 0.01	2.59	−1.94, 7.09
	6 months	1.10	−3.70, 6.02	−0.01	−0.04, 0.01	1.33	−3.15, 5.78
	12 months	1.38	−3.16, 6.00	−0.03	−0.05, 0.00	0.57	−4.00, 5.20
Group^2^	Usual care	REFERENCE		REFERENCE		REFERENCE	
	Intervention	−0.73	−6.02, 4.63	−0.01	−0.05, 0.03	−2.59	−7.77, 2.49
Group: Time^3^	Baseline # Usual care	REFERENCE		REFERENCE		REFERENCE	
	3 months # Intervention group	**7.69**	**2.29, 13.11**	0.02	−0.01, 0.05	2.22	−3.16, 7.57
	6 months # Intervention group	**7.01**	**1.46, 12.61**	0.02	−0.00, 0.07	1.67	−3.66, 6.90
	12 months # Intervention group	**5.89**	**0.53, 11.19**	**0.04**	**0.01, 0.07**	**6.44**	**0.99, 11.83**
Evidence ratio^4^	12 months # Intervention group > 0	60.33		609.17		98.72	
Posterior probability^5^	12 months # Intervention group	0.98		1.00		0.99	
Intraclass correlation coefficient	-	0.44		0.48		0.49	

-: not used in model

Evid. Ratio: Evidence ratio

Mean est: Estimation of mean

ICC: Intraclass correlation coefficient

m: months

Post. Prob: posterior probability

^1^Parameter estimates are the mean change from baseline for the control group at each follow up time point

^2^Parameter estimate compares the mean outcome at baseline between intervention and control

^3^The key parameter of interest, representing the difference in mean change from baseline between intervention and control

^4^Evidence ratio (pd/1-pd)

^5^The probability the parameter is greater than zero for positive estimates, or less than zero for negative estimates (the probability of direction (pd))

Bolded values indicate statistical significance

This suggests that the increase in percentage energy from core foods peaked at 3 months (7.7, 95%CI 2.3–13.1), following the intensive dietary intervention phase for the intervention group, with a smaller increase at 6 months (7.0, 95%CI 1.5–12.6). Percentage energy was shown to be higher among participants for each additional year of age by approximately 0.6% (0.6, 95%CI 0.1–1.0), and lower in men (−4.6, 95%CI −8.9, −0.0).

Quality-of-life scores ranged in value from 0 to 1, with −0.01 (0.07) mean change from baseline to 12-months in the UC group and 0.02 (0.05) for the intervention group. The estimated adjusted difference in QOL mean change between the groups was significantly higher in the intervention group compared to baseline (0.04, 95%CI 0.01–0.07). The posterior probability of > 99% indicated greater QOL increase in the intervention group compared to UC. QOL scores were higher in those with household incomes above an estimated $1,000 per week (before tax), compared to those with household incomes of less than $700 per week.

The mean change in PAM score between baseline and 12-months was 7.04 (14.3) for the intervention group and 0.72 (12.3) for the UC group. The estimated adjusted difference in mean change between the groups (intervention minus UC) was 6.44 (95%CI 0.99–11.83), with a 99% probability that the change was positive, indicating greater improvement in the intervention group. Results for the proportion of people attaining a 5% or greater weight loss and health literacy measures are reported in Table 4. For proportions of participants who had ≥ 5% weight loss at 12-months, there was a 94% posterior probability that there was a higher proportion in the intervention group, with an odds ratio of 12.55 (95%CI 0.53, 907).

**Table 4:** Results from outcomes using data only from baseline and 12-months, namely ≥ 5% weight loss and health literacy

		≥ 5% weight loss (baseline to 12-months)		Health literacy (AAHLS)		Electronic health literacy (eHEALS)	
Variable	Level or statistic	Odds ratio	95% CI	Mean est	95% CI	Mean est	95% CI
Intercept		0.00	−0.00, 2.66	18.45	12.54, 24.41	20.48	6.30, 34.49
Age	Years	1.08	0.92, 1.28	0.09	-0.01, 0.18	0.07	−0.15, 0.30
Gender	Female	REFERENCE		REFERENCE		REFERENCE	
	Male	0.98	0.16, 6.69	−1.00	−2.06, 0.05	−2.14	−4.66, 0.35
Education	Year 12 or less	REFERENCE		REFERENCE		REFERENCE	
	Trade or vocation	3.10	0.37, 28.50	**1.48**	**0.28, 2.66**	1.58	−1.26, 4.42
	University or higher	4.31	0.81, 26.31	**1.58**	**0.36, 2.79**	**3.18**	**0.26, 6.08**
% E core food	Percentage	0.98	0.91, 1.05	-	-	-	-
SR overweight or obese	No	REFERENCE		-		-	
	Yes	2.29	0.52, 10.91	-	-	-	-
Time^1^	Baseline	-		REFERENCE		REFERENCE	
	12 m	-	-	0.55	−0.50, 1.62	**2.25**	**0.21, 4.40**
Group^2^	Usual care	REFERENCE		REFERENCE		REFERENCE	
	Intervention	12.55	0.53, 906.87	0.61	−0.64, 1.87	0.00	−2.86, 2.88
Group # Time^3^	Baseline # Usual care	-		REFERENCE		REFERENCE	
	12 m # Int	-	-	0.52	−0.72, 1.76	−1.26	−3.73, 1.15
Evid. Ratio	12 m # Int. group > 0	16.7		3.99		0.18	
Post. Prob	12 m # Int. group	0.94		0.80		0.15	
ICC				0.51		0.64	

Mean estimates are derived from Bayesian mixed models, using MCMC sampling with four chains of 10,000 iterations and random intercepts for individuals and primary care practices, and accounting for any differences between groups at baseline. ≥ 5% weight loss is fitted with a binary outcome

-: not used in model

% E: Percentage energy from core foods

Evid. Ratio: Evidence ratio

Mean est: Estimation of mean

ICC: Intraclass correlation coefficient

m: months

Post. Prob: posterior probability

SR: Self-reported condition

^1^Parameter estimates are the mean change from baseline for the control group at each follow up time point

^2^Parameter estimate compares the mean outcome at baseline between intervention and control

^3^The key parameter of interest, representing the difference in mean change from baseline between intervention and control

Bolded values indicate statistical significance

No substantive change was seen in either health literacy scale, however those with a trade or vocational qualification had a higher AAHLS (1.48, 95%CI 0.28–2.66) compared to those with a year 12 or below education. Those with a university education or above had higher estimates of health literacy in both scales used (AAHLS: 1.58, 95%CI 0.36–2.79, and eHEALS: 3.18, 95%CI 0.26–6.08 respectively). The eHEALS indicated greater improvement in electronic health literacy in both groups at 12 months (2.25, 95%CI 0.21–4.40), although this finding was not consistent with the AAHLS.

## Discussion

The current study is the first personalized MNT telehealth intervention delivered by dietitians in primary care specifically for rural Australians identified as being at an elevated risk of CVD. Results presented here are an exploratory secondary outcome analysis and cannot be described as definitive. Overall, the posterior probabilities indicated that the intervention demonstrated a sustained significant improvement in the percentage of energy from core foods in the intervention group compared to the usual care control group at 12 months. Similarly, significant improvements in QOL and patient activation measures were identified at 12 months. Additionally, more people were able to achieve a 5% weight loss or greater when randomized to the intervention with the APD. This is important given there was no active intervention during the 6–12-month time period.

The baseline characteristics of participants in this study indicated that people at risk of CVD in this rural setting have complex risk profiles, which is reflective of the wider population. Characteristics included participants being more likely to be male, over 60 years, with lower incomes (< $1000/week) and higher blood pressure or cholesterol levels. Further, many participants initially demonstrated dietary patterns that did not align with national guidelines and chronic disease recommendations [13, 16]. Dietary intakes were characterised by a higher than recommended intake of energy-dense, nutrient poor discretionary foods and lower than recommended intake of nutrient-dense, core food groups, which is consistent with Australian population data more broadly [55, 56]. Notable differences found in this population that are not reflected in other at-risk population groups include the lower levels of overweight and obesity compared to Australian population data [55, 57], and participants in this study who lived alone had significantly higher diet quality than those who reported living with a partner [55, 57, 58]. Despite these differences, the characteristics of the population suggests the potential transferability of the HealthyRHearts intervention across similar at-risk populations.

The between-group difference in percent energy from core foods (between 5.9 and 7.7% of energy) was sustained 12 months following commencement of the trial, indicating that MNT can also sustain improved diet quality in this population once the intervention ceased. This finding is important, as previous research has shown that in the long term, healthier dietary patterns (i.e. those higher in core and lower in non-core food intakes) are associated with longer life expectancy [59], reduced healthcare costs [60] and a lower total risk of CVD [61]. Interventions resulting in healthier dietary patterns are associated with significantly reduced incidence of CVD events [62], CVD-related mortality [62, 63] and CVD risk factors such as blood pressure, LDL-cholesterol and triglycerides [64]. Clinically, a 6% reduction in energy-dense, nutrient poor, non-core food intakes is the equivalent of a reduction of 1 serve per day (522kJ/day [~ 125kCal/day]) for an average Australian who consumes 8700kJ/day (~ 2100kCal/day) [65]. A modelling study has shown that this relatively small, but long-term change of reducing non-core food intake by one serving per day in Australia would prevent the incidence of ischaemic heart disease by between 2198 and 17,439 cases annually, depending on the type of non-core food reduced [66]. This represents a potential positive impact if the MNT intervention were rolled out more broadly.

The results of the current study indicate that personalised MNT can not only result in significant increases in percentage energy from core food groups, but also significantly improve patient activation and health-related QOL. These improvements could impact many areas of a participant’s healthcare experience, with higher PAM scores associated with greater engagement in preventative health behaviours, reduced hospital admissions and health care service utilisation, improved health care cost-effectiveness, and better clinical outcomes, while higher EQ-5D scores indicate improved perceived health and well-being [67–71]. Dietitians delivering the HealthyRHearts MNT intervention received training in patient-centred behaviour change counselling techniques, which is highly likely to have contributed to improved participant activation and QOL measures. Patient-centred care has been associated with improved patient self-efficacy, which has in turn been found to be a significant predictor of patient activation and improved QOL [72–74]. The results of the HealthyRHearts trial highlight the role dietitian MNT consultations can have in improving multiple aspects of participant health, beyond dietary counselling.

Overall, there is limited evidence on the role of MNT on QOL [75, 76]. This study shows some promise that MNT delivered by an APD improves QOL, especially in patients at an elevated risk of CVD [76] however, compared with other dietary interventions reporting meaningful change in EQ-5D score, the change threshold in this study was lower (0.04 compared with ≥ 0.08) [77, 78]. Two factors that could have contributed to the change in QOL observed in this study were the easing of social distancing restrictions with the completion of the COVID-19 lockdown laws, which resulted in increased freedom and access to healthcare for some participants completing this study. Further, feelings of happiness and pride amongst those achieving their health goals as a consequence of the MNT intervention may have impacted QOL scores [79–83].

The current study also contributes to our limited understanding of the most effective mode of delivery for telehealth MNT interventions to sustainably improve diet-related CVD risk factors. Results suggest that shorter, more frequent consultations, equating to a total of 2-h contact with a dietitian over a 6-month period is effective for improving diet quality, patient activation and QOL. Further, statistically significant improvements in these outcomes were measured 6-months after the last dietitian consultation, indicating this mode of delivery can have lasting effects. When measured against other comparable research studies, five telehealth consultations delivered over 6-months to a total of two hours contact would be classified as a medium intensity intervention [84]. However, under the current Australian Government Chronic Disease Management plan (CDMP), and upcoming General Practitioner Chronic Condition Management Plan (GPCCMP) this intensity may not be feasible, as this plan only subsidises five consultations across multiple allied health professionals over a 12-month period [85, 86]. Therefore, for this intervention to be translatable to a primary care setting in Australia, an increase in subsidised appointments under the current CDMP and upcoming GPCCMP would be required, or more research would need to be done to investigate whether less contact time resulted in comparable improvements in patient outcomes.

While addressing health literacy is a priority for engaging participants in healthier lifestyle behaviours, including diet quality, the current study did not show significant improvements in health literacy even though significant improvements in other health-related outcomes was observed. Few interventional studies have assessed health literacy changes in nutrition interventions [87, 88], and additionally, no individual nutrition counselling interventions have shown significant improvements in health literacy [88]. Further research is required to assess the impact of MNT on overall health literacy in intervention studies.

This study obtained self-reported measures from participants at four separate time points and data from practices that pertained to heart health assessments at baseline and annual visits. However, by 12 months, there were many instances where data was obtained from only one of these sources, or from neither. While 80% of participants provided data for at least one study measure at 12-months, it was variable as to whether it was provided directly to the GP, or to the researchers. Response rates for survey measures varied between 49 and 54% for usual care and intervention groups, and 41–43% for dietary surveys. The reduction in response rates for the dietetic survey may have been because this survey was delivered from an external website and listed second in an email describing tasks to be completed. It is difficult to provide comparison response rates, as there are few similar studies, in terms of the population, time points at which data was collected and the type of intervention. One longitudinal study, the Australian Rural Mental Health Study [89], which had a focus on rural communities, reported a 64% survey response rate one year later. Similarly, a primary care study delivered in Arizona, United States of America, which also provided surveys electronically at 12-months reported that of the 253 people finishing their intervention, 70% completed both baseline and 12-month follow-up questionnaires [90]. It is possible that the tasks associated with this study over the various time points, namely visiting their GP, providing a fasting blood sample and completing health and nutrition surveys electronically was beyond what was reasonable to be asked. Further research is required to determine the extent of tasks considered acceptable for this and similar rural cohorts.

### Strengths and limitations

This study was the first telehealth, MNT intervention implemented by APDs and conducted in a rural setting. Additionally, this study was ‘place-based’, meaning it was informed by rural researchers, APDs, GPs and communities who live and work in the region that the trial was conducted. Further, this pragmatic trial was conducted in a primary care setting and in line with current Australian public healthcare system funding and referral approaches, although this model would limit consultations with other allied health in an annual funding cycle in the real-world setting.

Limitations of the current study include that the intervention was affected by COVID-19 and its effects on the GP workforce [91, 92], which contributed to the target sample size not being met. To address this limitation a Bayesian statistical model was used in line with recommendations [52, 93]. Additionally, this sample reported that only 42% completed 12-month dietary measures. All self-reported data (including dietary intake data) was used in the analysis to preserve the sample size, which included data with indications of over and under-reporting. Our modelling approach (mixed effects regression models) was consistent with intention-to-treat analyses as the model does not drop individuals from the analysis if they have missing follow-up data. This is a valid approach under the assumption that any missing follow-up data is missing at random. The baseline data contributes to the between group estimate of the difference in the change scores, which is a weighted average of the within and between group components. Also, as a self-administered tool, the dietary intake tool used (AES-Heart) is subject to self-reporting errors [94]. Dietary reporting has also been shown to be influenced by participation in interventions and the results from this study should be interpreted with these limitations in mind [95].

## Conclusions

Findings indicate that the provision of five MNT telehealth consultations by an APD, over a six-month period, are sufficient to clinically and statistically significantly improve intake of nutrient-dense core foods in a population of rural Australians at moderate to high risk of CVD. Findings further support additional benefits, including significant improvements in quality of life and patient engagement in managing their own health. The total of two-hours of MNT from the combined five telehealth consultations delivered by APDs was consistent with funding currently by Medicare, Australia’s public healthcare system. Although this may not be achievable given the current funded healthcare plan would normally be shared with other allied health providers. The program is currently being reviewed. Improvements in nutrient-dense, core food intakes, QOL and patient engagement were observed at 12-months, but further research is needed to evaluate whether these changes persist in the longer term, or further counselling is required to sustain results beyond 12 months.

## Supplementary Information

Supplementary Material 1.

Supplementary Material 2.

Supplementary Material 3.

Supplementary Material 4.

## Data Availability

Available upon reasonable request to the corresponding author.

## Abbreviations

95%CI: 95% Confidence interval
AAHLS: All Aspects of Health Literacy Scale
AES-Heart: Australian Eating Survey – Heart version
APD: Accredited Practising Dietitian
CVD: Cardiovascular disease
CDMP: Chronic disease management plan
COM-B: Capability, opportunity, motivation for behaviour change
eHeals: EHealth literacy scale
EQ-5D: Health-related quality of life measure
ESS: Effective Sample Size
Evid. Ratio: Evidence ratio
GP: General practitioner (primary care physician)
GPCCMP: General practitioner chronic condition management plan
HNECC PHN: Hunter New England central coast primary health network
ICC: Intraclass correlation coefficient
IQR: Interquartile range
LDL: Low-density lipoprotein cholesterol
MCMC: Markov chain Monte Carlo
MM or MMM: Modified Monash / modified Monash model
MNT: Medical nutrition therapy
PAM: Patient activation measure
PE: Percentage energy
PNQ: Personalised nutrition questionnaire
Post. Prob.: Posterior probability
QOL: Quality of Life
SD: Standard deviation
UC: Usual care
