# Supplementary material for: The effect of telehealth-based medical nutrition therapy on cardiovascular disease risk factors in a rural population: a secondary analysis of outcomes related to nutrition, health and well-being from the healthy rural hearts randomised controlled trial
Source: Int J Behav Nutr Phys Act. 2025 Oct 13;22:126. doi: 10.1186/s12966-025-01819-3 (PMC12519800; doi:10.1186/s12966-025-01819-3)
Supplement: Supplementary file 1 — Supplementary Material 1. [file 12966_2025_1819_MOESM1_ESM.pdf]

Australian Eating Survey

PARTICIPANT NAME HERE

# Your Dietary Analysis Report

**Date completed**

15th November 2021

On this page:

**Your intake of Healthy nutrient-rich foods vs Unhealthy nutrient-poor foods**

**Your intake of foods contributing to your heart health**

**Your intake of Carbohydrate, Protein, Fat and Alcohol**

**Your intake of Fibre, Vitamins and Minerals**

This report compares your usual dietary intake to Australian dietary recommendations and recognises areas of your diet which could be improve to maximise heart health.

**What's in this report?**

**Your AES Heart dietary feedback report summarises food and nutrient intakes:**

1. The first section provides feedback on how the foods you eat contribute to nutrient-rich energy intake and overall nutrient-poor intake. This information is displayed in two categories (based on the type of food): a) your healthy core food intake (e.g. vegetables; fruits; fish, chicken and other meats; milk, yoghurts and cheeses; and, grains and cereals), and b) your unhealthy foods (e.g. Sweetened drinks and packaged snacks) for sodium, added sodium is also displayed.
2. The second section summarises your intake of foods contributing to heart health (i.e., nuts, fats and oils, fish and seafood, vegetable proteins, fibre products and plant-based foods) and compared to recommendations
3. The third section summarises your nutrient intake and is separated into macronutrients (e.g. protein, total fat, saturated and trans fats, unsaturated fats, carbohydrate and alcohol) and micronutrients (e.g. fibre, vitamins and minerals). Your nutrient intake is compared to national nutrition recommendations based on your sex, age and for females, if you are pregnant and/ or breastfeeding.

For more information on how your Australian Eating Survey® report is generated, **click here** (https://australianeatingsurvey.com.au) .

## About this report

The food you eat helps to keep your heart healthy. Research shows that food affects heart health in a number of ways. These are:

- Eating habits that include high amounts of foods high in added sugar and saturated and trans fats can increase "unhealthy" levels of LDL cholesterol.
- Adding other dietary components such as soluble fibre, foods enriched with plant stanols/sterols, soy protein and the correct balance of fats in the diet can further enhance heart health

The information contained in this report is designed for general purposes only. It will not take into account any pre-existing medical conditions or other individual circumstances (such as use of vitamin and/or mineral supplements or any food sensitivities or allergies). As a result, it may not be a complete representation of your individual circumstances and should not replace the advice of your medical practitioner or an **Accredited Practising Dietitian** (https://daa.asn.au/find-an-apd)

## Overall energy intake and foods contributing to your energy intake

### Average daily energy intake

Your daily energy intake is **6468 kJ/day**

### Healthy nutrient-rich foods and unhealthy nutrient-poor foods

Healthy foods, also called "core" foods, are needed by your body every day to provide essential nutrients and also help to keep your heart healthy. Unhealthy foods, also called "discretionary" foods, are energy-dense, nutrient-poor foods and drinks. The recommendation is to consume these foods only occasionally and in small amounts. These are foods that may be enjoyable, but are generally high in fat, sugar and sodium.

**Healthy nutrient-rich foods vs unhealthy nutrient-poor foods**

Fig.1 Foods in your diet contributing to your energy intake

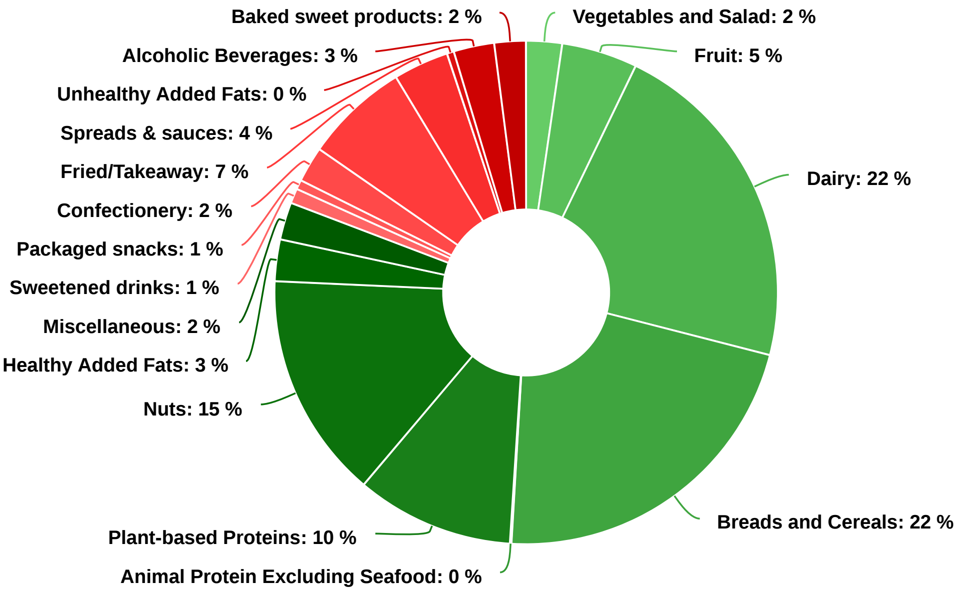


**Overall distribution of healthy and unhealthy foods as a proportion of energy intake**

| | |
|---|---|
| **Healthy nutrient-rich foods** | 80.81% |
| **Unhealthy nutrient-poor foods** | 19.19% |

**Ideal distribution of intake**

| | |
|---|---|
| **Healthy nutrient-rich foods** | 85-90% |
| **Unhealthy nutrient-poor foods** | 10-15% |

Fig.2: Contribution to overall sodium intake

Salt, also known as table salt, rock salt, sea salt and Himalayan salt, becomes sodium and chloride when absorbed by the body. It is a common ingredient in foods and the main source of sodium in our diet.

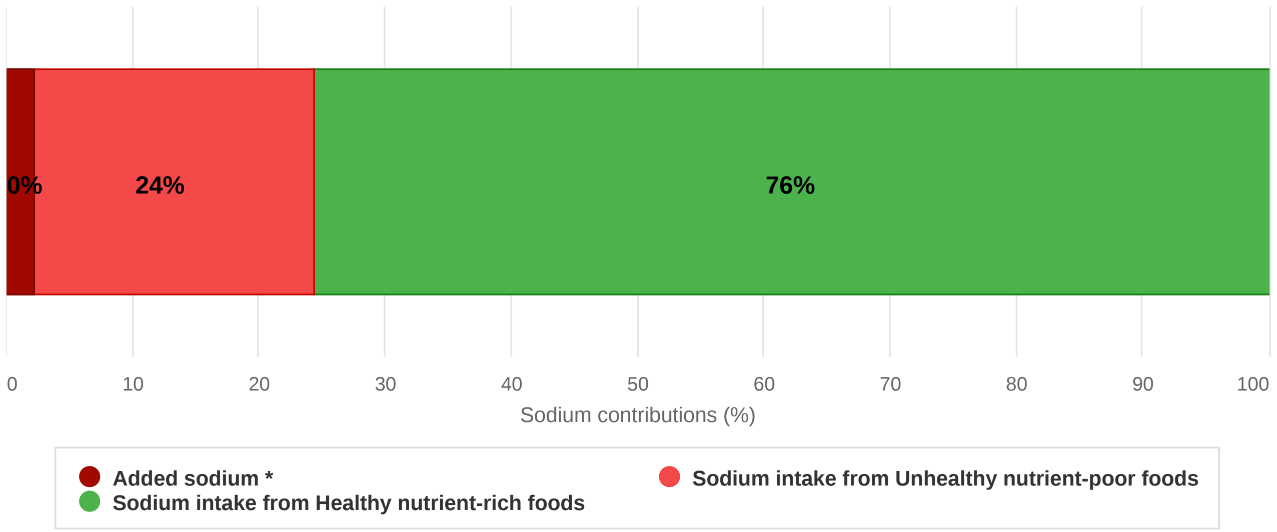


* Relates to sodium added when eating, cooking or preparing meals.

## Your intake of foods contributing to your heart health

Why are these foods good for heart health?

Extensive scientific research has shown the following foods help to reduce the risk of heart disease or a stroke. Having a diet with high frequency and variety of these foods can reduce LDL ("bad") cholesterol levels and provide protective benefits by increasing the amount of protective HDL ("good") cholesterol

Table 1: foods contributing to your heart health

| Foods | Your intake | Recommendation |
|---|---|---|
| Fruit | You consume 1 serves of fruit per day, comprising 4 different types of fruit per week. | 2 per day[1] and aim for different colours and types (i.e., citrus, berries, bananas, melons). |
| Vegetable | You consume 1.5 serves of vegetables per day comprising of 4 types of vegetables per week. | 5+ per day[1] and aim for different colours and types (i.e., leafy greens, zucchini, onions, carrots, cabbage). |
| Nuts | You consume plain nuts 0.9 times per day, comprising 1 types of nuts per week. | Aim for one serve of nuts per day[2] and a variety of unsalted nuts. One serve of nuts = 30g. |
| Fish & Seafood | You consume fish and seafood 0 times per week and usually have Other fish and seafood. | Aim for fish 2-3 times a week[3] and choose steamed, baked, pan-fried or grilled over deep-fried. One serve =100g cooked fish fillet. |
| Sterols | You consume 0.46g of sterols per day. | Between 2-3 grams per day[4]. For example, from foods with added sterols such as margarine, milk and weetbix. |

| Foods | Your intake | Recommendation |
| --- | --- | --- |
| Plant-based Proteins including legumes, lentils, beans and soy proteins | You consume plant-based proteins 0.7 times per day. | Aim for at least one serve from this group each day[3][5]. One serve = 150g (1 cup) of baked beans, lentils or other canned beans or 170g of tofu. |
| Dairy foods including milk, yoghurts and cheeses | You consume 2.6 serves per day and the type of milk you usually have is Reduced fat and the type of cheese you usually have is Reduced fat. | At least 4 serves per day (mostly reduced fat varieties)[1]. Choose mostly reduced fat varieties. One serve = 250ml milk or 2 slices of cheese, or 200g yoghurt. |
| Grains and cereals | You consume 3.1 serves of grains and cereals per day, with an average 2 types of products containing grains & cereals per week. | Aim to increase the variety of wholegrain foods that are rich in different fibre types (i.e., wholegrain breads, breakfast cereals, pasta, rice, cous cous, popcorn, flour) . One serve = 1 slice of bread, 1⁄2 cup cooked porridge, 1⁄2 cup cooked rice or pasta, 2/3 cup of wheat cereal flakes. |
| Red meat | You consume red meat 0 times per week, and the type of meat you usually eat is Don't eat meat. | Limit red meat consumption to less than 350g (1-3 meals)[3][6] per week. Avoid processed meat such as sausages, hot dogs, salami, ham, salted and cured meat, corned beef. |


1. Department of Health, Australia (https://www.eatforhealth.gov.au/food-essentials/how-much-do-we-need-each-day/recommended-number-serves-adults)
2. Better Health Vic (https://www.betterhealth.vic.gov.au/health/healthyliving/Nuts-and-seeds?viewAsPdf=true#:~:text=Recommended%20daily%20serving%20of%20nuts&text=This%20equal%20to%20about%3A,of%20peanuts%20or%20mixed%20nuts)
3. Heart Foundation Australia: Protein and heart health (https://www.heartfoundation.org.au/Heart-health-education/Protein-and-heart-health)
4. Heart Foundation Australia - Phytosterol-Stanols QA (https://www.heartfoundation.org.au/getmedia/d25f0d73-54b2-4399-a645-e0958c4dece4/Phytosterol-Stanols_QA_Professional_2017.pdf)
5. National Center for Biotechnology Information: Soy Protein (https://www.ncbi.nlm.nih.gov/pmc/articles/PMC1595159/#:~:text=Individuals%20need%20to%20consume%20about,a%20particular%20food's%20soy%20content)
6. Heart Foundation: Meat & Heart Healthy Eating (https://www.heartfoundation.org.au/getmedia/d5b9c4a2-8ccb-4fe9-87a2-d4a34541c272/Nutrition_Position_Statement_-_MEAT.pdf)


**Cooking tips to improve intake of your heart healthy foods:**

- Cook with cooking oils made from plants or seeds, including: olive, canola, peanut, sunflower, soybean, sesame and safflower.
- Use unsaturated fats in place of saturated fats (e.g. use avocado or table spread in place of butter in your sandwiches)
- 2-3 fish serves per week. Fish that is grilled, poached, steamed or pan-fried is preferred.
- Add a flavoured olive oil (or make your own) to salads as a dressing
- Replace red meat with plant-based protein foods, such as tofu, lentils and beans
- Limit red meat consumption to no more than three portions per week. (3 portions is equivalent to about 350–500g cooked weight).
- Snack on nuts, or add them to your salads and stir fries
- Use nut and seed butters, like peanut, almond, and tahini
- Switch wheat bran to oat bran
- Add in herbs and spices to flavour dishes instead of salt

# Your Nutrient Intake

This section summarises your nutrient intake and compares to the Australian Nutrient Reference Values for health.

## Macronutrients

Protein, carbohydrates and fat are all macronutrients and contribute to your kilojoule intake (energy intake). While alcohol is not a nutrient required by the body, it does contain kilojoules and so it contributes to your energy intake.

**Your macronutrient intake compared to the ideal intake range**

Fig.3 Macronutrients contribution to total daily energy intake (as % of energy intake)

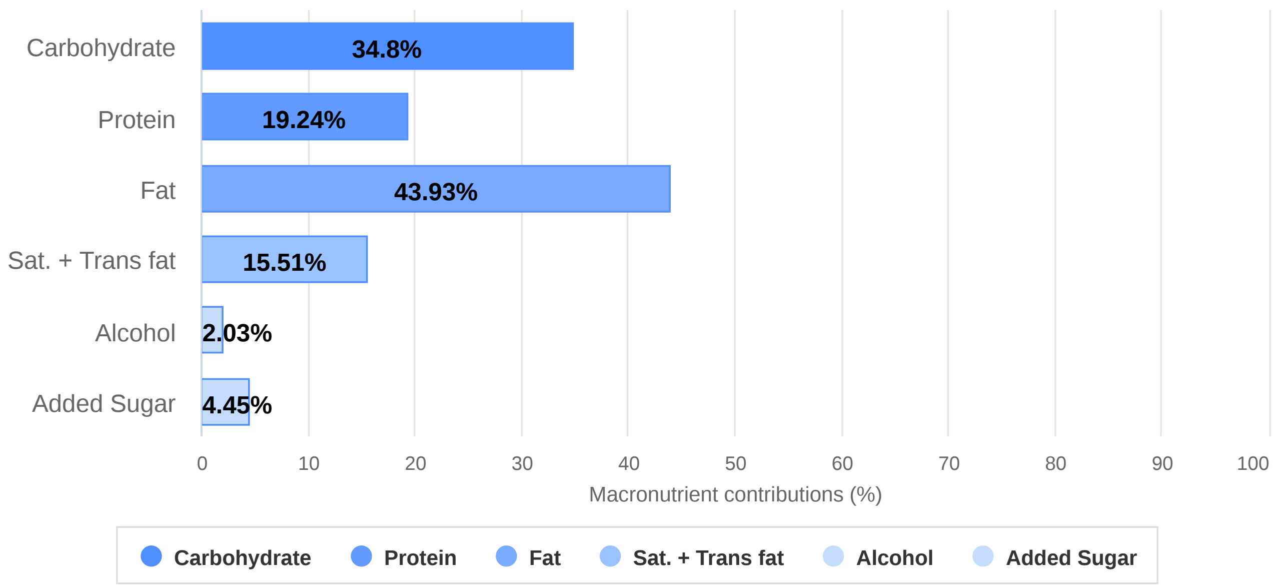


**Ideal intake ranges**

| | |
|---|---|
| **Carbohydrate** | 45-65% |
| **Protein** | 15-25% |
| **Fat** | 20-35% |
| **Sat. + Trans Fats** | <10% |
| **Alcohol** | <5% |
| **Added Sugar** | <10% |

This graph shows your intake of macronutrients as proportions of your total energy intake. A food intake that has carbohydrate, protein and fat intakes within the ideal ranges helps you to meet your requirements for general health. An increase in one macronutrient often leads to a decrease in others. If your nutrient intake is high in carbohydrate it tends to be lower in fat (and vice versa). Intakes higher in protein tend to be lower in carbohydrate and/or fat.

If you choose to consume alcohol, moderation is the key. Adult recommendations are for no more than two standard drinks per day. Children, adolescents (aged less than 18 years) and women who are pregnant, planning pregnancy or breastfeeding should not drink alcohol.

**Learn more about the importance of macronutrients**

**Carbohydrate:** Dietary sources of complex carbohydrates include grains and cereals (e.g. pasta, rice), breakfast cereals, breads, fruits, potato, corn and sweet potato, beans and lentils, dairy foods. Processed and refined carbohydrates are found in discretionary foods such as savoury snack foods (e.g. potato crisps, biscuits), some drinks (e.g. soft drink, fruit juice), confectionary and desserts. The fibre in wholegrain foods (i.e., brown rice, wholemeal flour, oats wholegrain bread and popcorn) can be good for your heart by reducing LDL cholesterol (known as bad cholesterol).

**Protein:** Rich sources of protein include lean meats, chicken, fish, eggs, nuts, dairy products and plant-based foods (e.g. lentils, chickpeas and beans).

**Fat:** There are four types of fat: saturated, trans, monounsaturated and polyunsaturated. Major sources of saturated and trans fats include fatty cuts of meat, full fat dairy foods, butter, cream, most commercially baked products (e.g. biscuits and pastries), most deep-fried fast foods, coconut and palm oil. Food sources of monounsaturated fats include margarine spreads (canola or olive oil-based), olive, canola and peanut oils, avocado, and nuts such as peanuts, hazelnuts, cashews and almonds. Food sources of polyunsaturated fat include oily fish (e.g. salmon, tuna, sardines), margarines and oils made from safflower, sunflower, corn or soy, and nuts such as walnuts and brazil nuts, and seeds.Not all fats are unhealthy. Unsaturated fats are the ‘healthy’ fats, they help to keep your cholesterol levels in the healthy range. Also try to avoid foods high in saturated fats (such as butter, coconut oil, cakes, pastries, pies, fat on meat, cream) and trans fats (such as biscuits, deep-fried foods and take- away foods). These fats can raise our blood cholesterol levels which is one of the risk factors for heart disease.

## Micronutrients and Fibre

Micronutrients are the vitamins and minerals that your body requires. Although the exact micronutrient requirements will vary from person to person, recommendations are made based on age, gender and life stage (ie pregnancy or breastfeeding). These recommendations can be used to determine whether your current food intake contains sufficient amounts of these key micronutrients.

**Your micronutrient and fibre intake compared to the ideal intake range**

Fig.4 Micronutrient and fibre intake

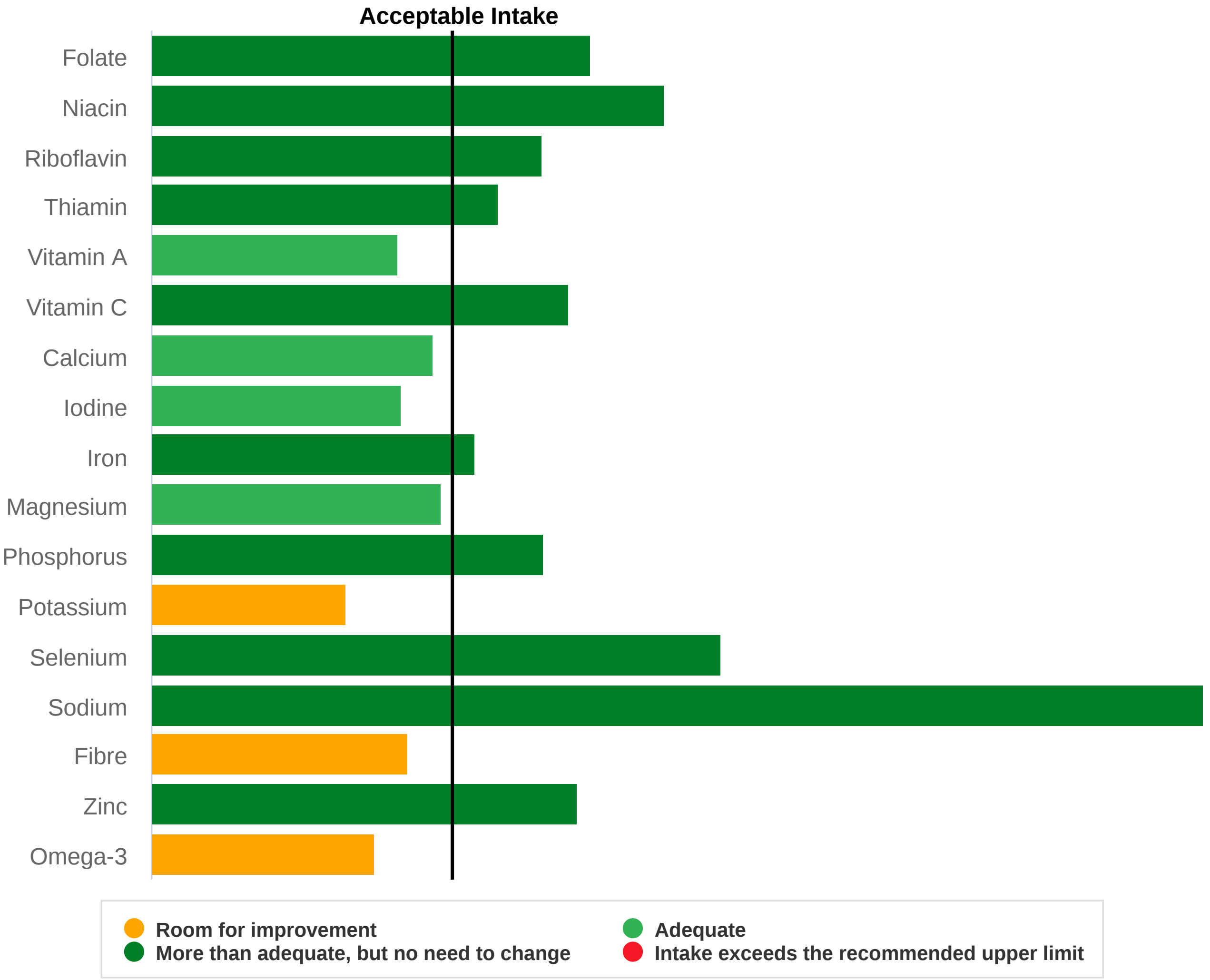


The graph above shows your micronutrient and fibre intake compared to the ideal intake range (i.e. Recommended Dietary Intake or Adequate Intake) for each nutrient.

For each nutrient on the graph above:

- An orange bar indicates that your usual intake for that nutrient is low and trying to eat more foods higher in this nutrient will help you reach the recommended intake.
- A light green bar indicates that your usual intake for that nutrient is in the target range but you could eat more foods that are high in this nutrient.
- A dark green bar indicates that your usual intake for that nutrient is adequate and there is no need to change.
- A red bar indicates that your usual intake for that nutrient is above the recommended limit and you should aim to cut back on foods high in this nutrient to avoid health problems. Not all nutrients have an upper limit.

**Your micronutrient and fibre intake based on your usual eating patterns:**

| Nutrient | Intake |
|---|---|
| **Vitamins** | |
| Folate | 585.1 ug/day |
| Niacin | 23.9 mg/day |
| Riboflavin | 1.4 mg/day |
| Thiamin | 1.3 mg/day |
| Vitamin A | 574.8 ug/day |
| Vitamin C | 62.5 mg/day |
| **Minerals** | |
| Calcium | 1221.4 mg/day |
| Iodine | 124.8 ug/day |
| Iron | 8.6 mg/day |
| Magnesium | 308.8 mg/day |
| Phosphorus | 1306.7 mg/day |
| Potassium | 1819.3 mg/day |
| Selenium | 113.7 ug/day |
| Sodium | 1960.8 mg/day |
| Fibre | 21.3 g/day |
| Zinc | 11.3 mg/day |
| Omega-3 | 67 mg/day |

Please note: your micronutrient analysis does not include any vitamin and/or mineral supplements that you may currently take.

### Common questions regarding micronutrient and fibre

**Do I need to take a vitamin and/or mineral supplement?**

This will depend on your situation. The nutrient analysis provided above does not account for any vitamin and/or mineral supplements that you may be taking currently nor any pre-existing medical condition or allergies. The Australian Eating Survey® is a validated tool for measuring dietary intake, but it asks you only about foods that are most commonly eaten in Australia.

If your analysis revealed your usual food intake is inadequate in one or more micronutrients, then try to increase your intake of foods that are good sources of those nutrients. If you need more help you could discuss the results from your Australian Eating Survey® with your doctor or an Accredited Practising Dietitian before taking a supplement. Simple changes to the foods that you usually eat will improve your nutrient intakes. Sometimes a supplement is required and your dietitian or doctor can provide you with the appropriate advice.

**How do I improve my intake of vitamins, mineral and fibre?**

As a guide, you may need to consume more of the foods that are good sources of the micronutrients and fibre that have been flagged in orange and light green in your graph above, and then cut back on those nutrient sources that appear in red. The table below contains general information about these nutrients, including the key food sources.

| Nutrient | Food sources |
|---|---|
| **Iodine** | Milk, cheese, yoghurt, seafood, sushi (containing seaweed), eggs, bread, prunes, corn. |
| **Thiamin (Vitamin B1)** | Wholemeal cereal grains, sesame seeds, soy beans and other dried beans and peas, wheatgerm fortified breakfast cereals, bread, yeast extracts including Vegemite® and Promite®, watermelon, yeast and pork. |

| Nutrient | Food sources |
|---|---|
| **Riboflavin (Vitamin B2)** | Milk, yoghurt, cheese, wholegrain breads and cereals, egg white, leafy green vegetables, mushrooms, Vegemite® and Promite®, meat, liver and kidney. |
| **Niacin (Vitamin B3)** | Lean meats, milk, eggs, wholegrain breads and cereals, tuna, salmon, nuts, leafy green vegetables. |
| **Folate (folic acid)** | Green leafy vegetables, legumes, seeds, liver, poultry, eggs, cereals and citrus fruits. Many cereal-based foods in Australia, such as bread and breakfast cereals, are fortified with folate. |
| **Vitamin C** | Fruit, especially citrus, pineapple, mango and pawpaw. Vegetables, especially capsicum, broccoli, Brussels sprouts, cabbage, spinach. |
| **Vitamin A** | Dark yellow, orange and dark green vegetables and fruit such as apricots, mango and rockmelon, carrots, sweet potato and pumpkin, spinach and broccoli. |
| **Zinc** | Meat, chicken, fish, oysters, legumes, nuts, wholemeal and wholegrain products. |
| **Iron** | There are two types of iron. **Haem iron** (which is more easily absorbed) - found in animal foods such as beef, chicken and fish and in liver and kidney. **Non-haem iron** - found in plant foods such as beans, nuts, lentils and leafy green vegetables. Vegetarian sources include iron-fortified breakfast cereals, flours and grains. Vitamin C and cooking boost iron absorption. |
| **Calcium** | Dairy foods, such as milk, cheese, yoghurt, canned salmon and sardines with the bones, fortified soy milks, leafy green vegetables, such as broccoli, bok choy, Chinese cabbage and spinach, brazil nuts, almonds and sesame seed paste (tahini). |
| **Phosphorous** | Lean meats, chicken, fish, milk, yogurt and cheese. |
| **Magnesium** | Tofu, soy beans, nuts, seeds, lean meat, spinach, barley, wheatgerm, brown rice, avocado, bananas, peanut butter and peas. |
| **Sodium** | Processed meats (e.g. ham, bacon, sausages), snack foods (e.g. biscuits, potato crisps), takeaway foods (e.g. pies, sausage rolls), canned foods (e.g. soups), and savoury cooking sauces (e.g. pasta and stir-fry sauces) and condiments (e.g. tomato sauce, mayonnaise). Breads and fat spreads, breakfast cereals and cheese can also be high in sodium but provide many other important nutrients. |
| **Potassium** | Most fruits and vegetables, particularly leafy greens, potatoes, tomatoes, pumpkin, legumes, bananas, oranges, dairy products, and nuts. |
| **Soluble fibre** | Fruits, vegetables, oat bran, barley, flaxseed, dried beans, lentils, peas, soy milk and soy products. |
| **Insoluble fibre** | Wheat bran, corn bran, rice bran, the skins of fruits and vegetables, nuts, seed dried beans and wholegrain foods. |
| **Selenium** | Brazil nuts, tuna, halibut, canned sardines in oil, shrimp, chicken, cottage cheese, eggs, baked beans, oats, brown rice. |
| **Omega-3** | Cold water fish such as salmon, mackerel and sardines, nuts and seeds such as linseed and walnuts, plant oils (flaxseed oil, soybean oil). |
