# Supplementary material for: The effect of telehealth-based medical nutrition therapy on cardiovascular disease risk factors in a rural population: a secondary analysis of outcomes related to nutrition, health and well-being from the healthy rural hearts randomised controlled trial
Source: Int J Behav Nutr Phys Act. 2025 Oct 13;22:126. doi: 10.1186/s12966-025-01819-3 (PMC12519800; doi:10.1186/s12966-025-01819-3)
Supplement: Supplementary file 4 — Supplementary Material 4. [file 12966_2025_1819_MOESM4_ESM.docx]

**Supplementary materials 2: Outcome measures**

| **Measure** | Baseline | + 2 weeks | + 4 weeks | 3 months | 6 months | 12 months |
| --- | --- | --- | --- | --- | --- | --- |
| **MNT consultations** | 1^st^ MNT consult  (30 min) | 2^nd^ MNT consult  (20 min) | 3^rd^ MNT consult  (20 min) | 4^th^ MNT consult  (20 min) | 5^th^ MNT consult  (30 min) |  |
| (Intervention group only) | 🗹 | 🗹 | 🗹 | 🗹 | 🗹 |  |
| **Pathology (fasting)** |  |  |  |  |  |  |
| Total cholesterol, LDL and HDL cholesterol, triglycerides, glucose & Hba1c | 🗹 |  |  | 🗹 | 🗹 | 🗹 |
| **Anthropometry & physiological measures** |  |  |  |  |  |  |
| *Measured by GP* |  |  |  |  |  |  |
| - Blood pressure | 🗹 |  |  |  |  | 🗹 |
| - Weight^†^ | 🗹 |  |  |  |  | 🗹 |
| - Waist circumference | 🗹 |  |  |  |  | 🗹 |
| - Height | 🗹 |  |  |  |  |  |
| *Self-report by participant* |  |  |  |  |  |  |
| - Weight^†^ | 🗹 |  |  | 🗹 | 🗹 | 🗹 |
| - Waist circumference | 🗹 |  |  | 🗹 | 🗹 | 🗹 |
| - Height | 🗹 |  |  |  |  |  |
| **Participant surveys** |  |  |  |  |  |  |
| - Demographics^†^ | 🗹 |  |  |  |  |  |
| - Global Health Scale (QOL) ^†^ | 🗹 |  |  | 🗹 | 🗹 | 🗹 |
| - Sleep disturbance | 🗹 |  |  | 🗹 | 🗹 | 🗹 |
| - Physical activity | 🗹 |  |  | 🗹 | 🗹 | 🗹 |
| - Patient Activation Measure^†^ | 🗹 |  |  | 🗹 | 🗹 | 🗹 |
| - eHEALS^†^ | 🗹 |  |  | 🗹 | 🗹 | 🗹 |
| - AAHLS^†^ | 🗹 |  |  | 🗹 | 🗹 | 🗹 |
| - AES-Heart^†^ | 🗹 |  |  | 🗹 | 🗹 | 🗹 |
| **Key:**  †: reported as an outcome  AAHLS: All Aspects of Health Literacy Scale  AES-Heart: Australian Eating Survey – Heart version  eHeals: eHealth Literacy Scale  QOL: Quality of Life (reported as EQ-5D) | | | | | | |

*Participant demographics*

Participants were asked to self-report co-morbidities, their health insurance status, education level, household income and living arrangements. Education, living arrangements and income categories were collapsed for reporting purposes, to protect anonymity of low response answers. Participants who that received a pension as their income were nominated a value based on their response to their living arrangements (i.e. a single person or couple) and according to Australian age pensions ^(1)^. As the whole population were expected to be based in a non-metropolitan place, questions about size of the town they lived in (or nearest town) were also asked. Participant age and sex were obtained from the CVD risk profile provided by each participant’s GP.

*Anthropometry*

At baseline, 3-, 6- and 12-months participants self-reported their height (cm), weight (kg), and waist circumference (cm) using instructions and reference videos provided by the research team. Body mass index (BMI) was calculated as weight (kg) / [height (m)]^2^. GPs were asked to provide blood pressure, as well as height and weight at baseline and 12-months, in the case that participants were not able to collect their own weight and to substantiate values provided. Two self-reported weight measures from baseline and 12-months were preferred over GP measures, unless a data entry error was suspected, as participants were instructed to take measures at a consistent time of day with similar clothing, which may not have been possible at a GP visit. In the case where only a baseline value existed, a self-reported value was preferred for consistency.

*Quality of life and sleep*

Quality of life and sleep questionnaires were administered at each time point from the PROMIS short-form measures for adult health profiles ^(7)^ .The PROMIS GLOBAL Health Scale v1.2 (13th April, 2018, Global 10) was used to measure quality of life. The Global 10 consists of 10 questions and asks participants to report their experience over the last 7-days. Estimated EQ-5D-3L was calculated from the Global Health Items according to the scoring calculation recommended for output using the paper-based version ^(8)^.

Sleep was assessed using the PROMIS short form Sleep Disturbance 4a. This questionnaire consisted of four items and was also administered using REDCap at all four timepoints of the study. From the sleep questionnaire a T-score was calculated ^(9)^, this involved rescaling the raw score into a standardised score, using a mean of 50 and a standard deviation of 10.

*Physical activity*

Four questions from the Active Australia Survey ^(10)^ were used to gauge the participant’s walking and vigorous activity in the last week and collected at each timepoint. A value was created by adding the total time spent walking to twice the amount of total time for vigorous activities.

*Patient Activation Measures (PAM)*

The PAM a validated 13-item questionnaire relating to motivation to engage in one’s own healthcare. It provides a score that is derived from a 100-point scale, and categorises individuals into four categories based on their knowledge, skills and confidence in managing their health: 1) ‘disengaged and overwhelmed’; 2) ‘becoming aware, but still struggling’; 3) ‘taking action’ and; 4) ‘maintaining behaviours and pushing forward’ ^(11)^. These categories relate to an individual’s perspective that: 1) ‘my doctor is in charge’; 2) ‘I could be doing more’; 3) ‘I am a part of my health care team’ and 4) ‘I’m my own advocate’ respectively. This questionnaire was repeated at all timepoints.

*Health literacy*

Two surveys were used to assess health literacy. General health literacy was assessed using the All Aspects of Health Literacy Scale (AAHLS) ^(12)^. Questions from three domains (functional, communicative and critical health literacy) were scored as; yes (3 points), maybe (2 points), no (1 point) or rarely (1 point), sometimes (2 points) and often (3 points). Questions FQ1 and FQ3 on functional health literacy were scored in reverse as published in the literature ^(12, 13)^. Functional health literacy question FQ2 was scored as the maximum value (3 points) as its scoring was not addressed in the literature. Questions on empowerment were not included in the scoring as they do not directly assess health literacy. Therefore, a total of 11-items were included with a maximum score of 33 points possible. Higher scores suggested higher health literacy.

Further, the eHealth Literacy Scale (eHEALS) was used to measure eHealth literacy, which is validated in older adults ^(14)^ The eHEALS is a brief screening tool where participants rate their level of agreement to 8-items on a 5-point Likert scale from strongly disagree (1 point) to strongly agree (5 points). Scores can range between 8 to 40 points, with higher scores suggesting higher eHealth literacy.

*Participant engagement in the program*

For the intervention group, data was collected on the number of MNT sessions attended and the average number of goals stated at baseline.

**References**

1. Australian Government. Age Pension - How much can you get Canberra, Australia: Commonwealth of Australia; 2024 [updated 20 Sept 2024]. Available from: <https://www.servicesaustralia.gov.au/how-much-age-pension-you-can-get?context=22526>.

2. Burrows TL, Hutchesson MJ, Rollo ME, Boggess MM, Guest M, Collins CE. Fruit and vegetable intake assessed by food frequency questionnaire and plasma carotenoids: a validation study in adults. Nutrients. 2015;7(5):3240-51.

3. Schumacher TL, Burrows TL, Rollo ME, Wood LG, Callister R, Collins CE. Comparison of fatty acid intakes assessed by a cardiovascular-specific food frequency questionnaire with red blood cell membrane fatty acids in hyperlipidaemic Australian adults: a validation study. Eur J Clin Nutr. 2016;70(12):1433-8.

4. Food Standards Australia New Zealand. AUSNUT 2011-2013 food nutrient database. FSANZ; 2014.

5. National Health and Medical Research Council. Australian Guide to Healthy Eating. Canberra: NHMRC; 2013 [updated 01 May 2017]; [cited 2021 03 Oct]. Available from: <https://www.eatforhealth.gov.au/guidelines/australian-guide-healthy-eating>.

6. Collins C, Burrows T, Rollo M. Dietary patterns and cardiovascular disease outcomes: An evidence check rapid review brokered by the sax institute (www. Saxinstitute. Org. Au) for the national heart foundation of australia. Accessed June. 2017;5.

7. Cella D, Choi SW, Condon DM, Schalet B, Hays RD, Rothrock NE, et al. PROMIS((R)) Adult Health Profiles: Efficient Short-Form Measures of Seven Health Domains. Value Health. 2019;22(5):537-44.

8. PROMIS Global Health Scoring Manual Northwestern University2018 [updated 9/16/2024]. Available from: <https://www.healthmeasures.net/images/PROMIS/manuals/Scoring_Manual_Only/PROMIS_Global_Health_Scoring_Manual_30Aug2024.pdf>.

9. PROMIS Sleep Scoring Manual Northwestern University2023. Available from: <https://www.healthmeasures.net/images/PROMIS/manuals/Scoring_Manual_Only/PROMIS_Sleep_Scoring_Manual_05Dec2023.pdf>.

10. Australian Institute of Health and Welfare. The Active Australia Survey: a guide and manual for the implementation, analysis and reporting. Canberrra: Australian Institute of Health and Welfare; 2003.

11. Hibbard JH, Stockard J, Mahoney ER, Tusler M. Development of the Patient Activation Measure (PAM): conceptualizing and measuring activation in patients and consumers. Health Serv Res. 2004;39(4 Pt 1):1005-26.

12. Chinn D, McCarthy C. All Aspects of Health Literacy Scale (AAHLS): Developing a tool to measure functional, communicative and critical health literacy in primary healthcare settings. Patient Educ Couns. 2013;90(2):247-53.

13. Barsell DJ, Everhart RS, Perrin PB. Refining the Factor Structure of the All Aspects of Health Literacy Scale. Am J Health Behav. 2020;44(2):118-28.

14. Chung S-Y, Nahm E-S. Testing reliability and validity of the eHealth Literacy Scale (eHEALS) for older adults recruited online. Computers, informatics, nursing : CIN. 2015;33(4):150-6.
